# Damage Control Surgery in Obstetrics and Gynecology: Abdomino-Pelvic Packing in Multimodal Hemorrhage Management

**DOI:** 10.3390/jcm14207207

**Published:** 2025-10-13

**Authors:** Stoyan Kostov, Yavor Kornovski, Angel Yordanov, Stanislav Slavchev, Yonka Ivanova, Ibrahim Alkatout, Rafał Watrowski

**Affiliations:** 1 Research Institute, Medical University Pleven, 5800 Pleven, Bulgaria; drstoqn.kostov@gmail.com; 2 Department of Gynecology, Hospital “Saint Anna”, Medical University—“Prof. Dr. Paraskev Stoyanov”, 9002 Varna, Bulgaria; ykornovski@abv.bg (Y.K.); st_slavchev@abv.bg (S.S.); yonka.ivanova@abv.bg (Y.I.); 3 Department of Gynecologic Oncology, Medical University Pleven, 5800 Pleven, Bulgaria; angel.jordanov@gmail.com; 4 Department of Obstetrics and Gynecology, University Hospitals Schleswig-Holstein, Campus Kiel, Arnold-Heller-Straße 3 (House C), 24105 Kiel, Germany; ibrahim.alkatout@uksh.de; 5 Department of Obstetrics and Gynecology, Helios Hospital Müllheim, 79379 Müllheim, Germany; 6 Faculty of Medicine, University of Freiburg, 79106 Freiburg, Germany

**Keywords:** damage control surgery, pelvic packing, obstetric hemorrhage, postpartum hemorrhage, placenta accreta spectrum, gynecologic surgery, massive hemorrhage, tranexamic acid, hemostatic agents, surgical emergencies

## Abstract

Damage control surgery (DCS) is a staged surgical strategy for rapid control of life-threatening bleeding, followed by physiological stabilization and delayed definitive repair. Abdomino-pelvic packing (APP)—placing compressive material within the pelvis and/or abdomen to tamponade bleeding—is a cornerstone of DCS as a temporizing measure to achieve hemostasis and stabilization in critically unstable patients. This narrative review synthesizes current evidence on DCS with a focus on APP—a technique historically developed in trauma and orthopedic surgery for exsanguinating pelvic bleeding but adaptable to gynecologic and obstetric emergencies. We outline the historical evolution, physiological basis, and stepwise protocol of DCS, adapted to specialty-specific conditions such as postpartum hemorrhage, placenta accreta spectrum, uterine rupture, and hepatic rupture in HELLP syndrome, as well as oncologic surgeries (debulking, exenteration, lymphadenectomy) and benign procedures (trocar-entry injuries in laparoscopy, presacral bleeding in sacrocolpopexy, and retroperitoneal hemorrhage in deep-infiltrating endometriosis). Modern adjuncts—including early tranexamic acid, topical hemostatic agents, and multidisciplinary coordination—have transformed packing from a last-resort maneuver into an integrated component of staged hemorrhage control. In OB/GYN, APP allows for successful hemostasis in 75–90% of cases, with significantly lower mortality rates than trauma surgery. In conclusion, APP as a potentially life-saving maneuver within DCS requires integration into standardized, institution-wide hemorrhage protocols in OB/GYN. Training, simulation, and guideline adoption are critical, particularly in resource-limited settings where advanced interventions for catastrophic bleeding are inaccessible.

## 1. Introduction

Perioperative and peripartum hemorrhage remain major causes of morbidity and mortality in gynecology and obstetrics (OB/GYN) [1,2,3]. Clinically significant bleeding is commonly defined as blood loss ≥ 1000 mL, the need for transfusion, or ≥25% loss of circulating volume; a loss of 30–40% typically precipitates cardiovascular instability and >40% is life-threatening [4,5]. Massive hemorrhage can occur unpredictably across the spectrum of gynecologic and obstetric practice, ranging from common, low-risk procedures such as cesarean section and hysterectomy [4,6,7] to highly complex oncogynecologic surgeries, including pelvic exenteration and cytoreductive surgery for advanced ovarian or cervical cancer [8,9,10,11]. In laparoscopy—currently the most frequent access route in gynecology—life-threatening hemorrhage can occur during the very first step: entry-related complications account for 60–80% of major vascular injuries, and catastrophic events have been reported even during basic procedures [12,13]. Life-threatening intrabdominal bleeding with hemorrhagic shock has been reported after coital injury [14,15,16]. Beyond procedure type, provider and center experience also matter: higher surgeon and hospital volumes correlate with lower in-hospital mortality after ovarian cancer surgery [17]. In contrast, even in highly complex procedures such as pelvic exenteration, death from exsanguination is uncommon in experienced hands [18,19].

Damage control surgery (DCS), initially conceptualized for military and civilian trauma by Rotondo et al. in 1993 [20], represents a staged surgical strategy prioritizing rapid hemorrhage control and physiologic stabilization over definitive repair in critically unstable patients [21,22,23,24]. The fundamental principles—abbreviated procedures, immediate control of bleeding/contamination, intensive care resuscitation, and planned reoperation—are adaptable to obstetrics and gynecology, although direct transfer of trauma algorithms requires careful consideration of specialty-specific differences [10,25,26,27,28]. Common causes of massive hemorrhage in OB/GYN include, among others, placenta accreta spectrum with pelvic sidewall bleeding, refractory uterine atony or traumatic postpartum hemorrhage (e.g., uterine rupture), severe trauma during childbirth, intractable pelvic oozing in patients with coagulopathy, vascular injury or damage of atypical neovascularizations during oncologic debulking, trocar-entry injuries in laparoscopy, presacral bleeding in sacrocolpopexy, and bleeding from retroperitoneal vessels in deep-infiltrating endometriosis [24,25,26,27,28,29,30].

Abdomino-pelvic packing (APP)—the placement of sterile, radiopaque, space-filling, deformable materials into the pelvis and/or abdomen to tamponade bleeding against bony or fascial planes when conventional measures fail—remains a cornerstone of DCS in selected scenarios [31,32,33,34]. Although formalized and widely studied in trauma and visceral surgical cohorts (e.g., unstable pelvic fractures, hepatic rupture) [31,32,33,34], APP was first reported in gynecologic oncology for massive pelvic hemorrhage [10] and has clear applications in several critical OB/GYN scenarios [6,7,35,36,37,38,39,40,41]. Materials are most commonly laparotomy pads or purpose-designed packs; in selected situations, other sterile space-occupying devices (e.g., balloon or prosthetic sizers) have been used following the same principle, but standard practice relies on pads or dedicated packs [35,37,40,42,43,44,45].

Many early techniques were developed when modern topical hemostatic methods were not available and when anatomical adjuncts and imaging were more limited. Importantly, neither papers from the 1990s [10] nor more recent reports consistently describe the measures that should precede APP, such as optimization of systemic hemostasis, use of local hemostatic agents, early and aggressive uterotonics, balloon tamponade, uterine compression sutures, arterial ligation, and selective arterial embolization [2,46,47,48].

The contemporary armamentarium provides surgeons with several improvements and aids for managing intraoperative and peripartum bleeding. These include blood management strategies, early tranexamic acid (TXA) administration [49,50,51,52], a broad range of hemostatic agents [3,44,48,53,54,55,56], and vascular control strategies—from internal iliac artery ligation [57,58,59] to selective endovascular arterial embolization or balloon occlusion [60,61]. While these modalities can de-escalate purely surgical maneuvers and limit tissue damage, catastrophic bleeding can still arise, even during seemingly low-risk procedures. For this reason, gynecologic and obstetric surgeons must remain proficient in the fundamentals of DCS, including timely application of APP [2,27,37].

Given that APP can be life-saving, in this narrative review, we present the history, principles, and techniques, as well as risks and complications, of APP as part of DCS adapted to modern OB/GYN practice.

## 2. History of Abdomino-Pelvic Packing

APP as a hemorrhage control technique was first described by James Hogarth Pringle in 1908, who managed severe hepatic bleeding with perihepatic packing combined with temporary occlusion of hepatic inflow—the latter now known as the Pringle maneuver [62]. In gynecology, Logothetopulos introduced a novel approach in 1926 for managing post-hysterectomy pelvic hemorrhage by inserting a sterile bag (X-ray cassette drape) filled with gauze into the pelvic cavity and drawing it through the vaginal vault to achieve compression of bleeding surfaces [63]. This technique, later termed Logothetopulos packing, underwent various modifications and was sometimes described as the “mushroom,” “parachute,” or “umbrella” pack due to its shape upon deployment [35]. Despite its innovative design, clinical adoption was limited by high rates of infection, with some series reporting infectious complications in up to 81.8% of cases [35,36,37,38,43]. During World War II and the Vietnam War, abdomino-pelvic packing did not gain wide acceptance among U.S. military surgeons due to concerns about infection and delayed complications. Nevertheless, civilian surgeons continued to use packing as a lifesaving measure, especially for hepatic injuries, and subsequent clinical experience supported its efficacy [22,23,31,64]. Renewed interest in the technique emerged by the late 20th century, with Stone et al. describing improved survival for coagulopathic abdominal trauma patients with abbreviated packing procedures. They packed the abdomen with 4–17 laparotomy pads to obtain tamponade, and the abdomen was closed under tension without drains or stomata [65]. The rebirth of APP came in 1993 with the landmark paper by Rotondo et al., which demonstrated a seven-fold improvement in survival (from 11% to 77%) among patients with combined visceral and major vascular injuries when applying the damage control concept. These authors were also the first to introduce the term “damage control,” which later evolved into “DCS” [20]. Since then, both the concept and terminology of damage control surgery have become integral to the guidelines of most trauma centers [32] and have been incorporated into OB/GYN-specific practice [2,7,9,25,27].

## 3. Physiological Basis and Core Principles of DCS in OB/GYN

DCS is a staged operative strategy for critically unstable patients that prioritizes rapid hemorrhage control and contamination control, followed by ICU resuscitation and planned reoperation for definitive repair [20]. The central objective is to interrupt the self-perpetuating progression toward the “lethal triad” of hypothermia, acidosis, and coagulopathy, which markedly increases mortality in exsanguinating patients [66]. Earlier, Kashuk and colleagues described the related “bloody vicious cycle,” highlighting how hemorrhage, cellular shock, and tissue injury reinforce one another and exacerbate bleeding [67].

Each component of the triad worsens the others and amplifies ongoing blood loss:

- *Hypothermia* (often <35 °C) impairs platelet function and coagulation enzyme activity, reduces cardiac performance, and shifts the oxyhemoglobin dissociation curve to the left, compromising tissue oxygen delivery [68,69,70].
- *Acidosis* (e.g., pH < 7.2 or base deficit > 8 mEq/L) results from tissue hypoperfusion and lactate accumulation; it directly inhibits multiple steps in the coagulation cascade and depresses myocardial function [24,71].
- *Coagulopathy* in this context is typically diffuse and non-mechanical (oozing from raw surfaces and venous plexuses) [65]. The diagnosis of coagulopathy is mainly clinical by observing generalized non-surgical bleeding from wounds, vascular access sites and others. Laboratory tests do not always confirm coagulopathy in critically injured patients. While abnormalities in standard labs (e.g., prolonged PT/aPTT, thrombocytopenia, hypofibrinogenemia) can support the diagnosis, clinical judgment remains central because conventional tests may not be immediately available, and—even when available—may fail to reflect real-time or clinically significant coagulopathy in critically injured patients [25,66,71,72].

In practice, DCS is considered when the patient’s physiology and operative course indicate that a prolonged definitive procedure is unsafe [25,27,32,66,69,73].

Common physiologic and resuscitative red flags include

- Massive transfusion requirements (e.g., ≥10 units PRBC or ≥5000 mL blood products within 24 h);
- Persistent hypotension (SBP < 90 mmHg) despite active resuscitation;
- Severe metabolic derangement (e.g., pH < 7.2, base deficit > 8 mEq/L);
- Hypothermia (<35 °C) and clinical coagulopathy (diffuse bleeding);
- Depressed sensorium (e.g., Glasgow Coma Scale decline) as a global hypoperfusion marker in hemorrhagic shock.

Operative and injury pattern red flags that commonly prompt an abbreviated strategy include [24,25,69]

- Excessive intraoperative fluid/blood product replacement (e.g., >12,000 mL crystalloids/blood products);
- Prolonged operative time in an unstable patient (e.g., >90 min without clear hemostatic control);
- High injury burden (e.g., ISS > 35) or combined injuries (major abdominal vascular and visceral injuries);
- Pelvic trauma with major vascular involvement;
- Inability to achieve tension-free primary fascial closure due to edema/packing, mandating temporary closure.

Once the decision for DCS is made, the operative strategy consists of four sequential phases [20,25,66]:

1. **Abbreviated laparotomy** focused solely on hemostasis (e.g., APP, rapid vascular control);
2. **Temporary closure** to allow for re-exploration;
3. **Intensive care unit (ICU) resuscitation** to reverse hypothermia, acidosis, and coagulopathy;
4. **Planned reoperation** for pack removal and definitive repair once physiological stability is achieved.

## 4. Topographic Typology of Packings in OB/GYN

A topographical classification of APP techniques provides a practical framework for guiding surgical decision-making and serves educational purposes in obstetrics and gynecology. By categorizing packing strategies according to the anatomical site of application, surgeons can tailor interventions to the presumed source of hemorrhage. The principal categories include those outlined below.

*Vaginal packing*—This is primarily used for persistent vaginal or cervical bleeding, such as from lacerations, episiotomy sites, or cervical tears occurring during childbirth or in the postoperative period. This technique remains relevant as a first-line mechanical tamponade for focal bleeding and is supported by its inclusion in recommended interventions for postpartum hemorrhage (PPH) [47]. Vaginal packing is not recommended in cases of PPH resulting from uterine atony [2].
*Uterovaginal packing*—This is traditionally applied in managing PPH secondary to uterine atony when pharmacological and other first-line methods fail. While classic gauze packing was historically standard, its use is increasingly supplanted or augmented by intrauterine balloon tamponade devices (e.g., Bakri balloon, condom catheter), which offer more controlled and quantifiable pressure with reduced risks of infection and migration [47]. Recent reviews highlight the role of these devices in reducing the need for surgical escalation [39].
*Vagino-pelvic packing*—This is indicated for uncontrolled pelvic bleeding after hysterectomy, especially when retroperitoneal hemorrhage or bleeding from the vaginal cuff is suspected. The technique, often referred to as Logothetopulos packing after its initial description in 1926, involves retrograde placement of sterile packs through the vaginal vault into the pelvic cavity to achieve tamponade of deep vascular structures or diffuse oozing surfaces [38,63].
*Pelvic packing*—This is performed directly within the pelvic cavity via laparotomy to control diffuse venous oozing, arterial bleeding from parametrial dissection, or injury to branches of the internal iliac system. Variants such as the “Mikulicz technique” involve layered placement of laparotomy pads to maximize compression against the bony pelvis and fascial planes [9,10,11]. This approach is documented in case series involving gynecologic oncology procedures, where rapid hemorrhage control is critical [9].
*Abdominal packing*—This is utilized for uncontrolled hemorrhage originating in the upper or middle abdomen, such as from liver or splenic injury, major vascular lesions, or diffuse peritoneal bleeding during extensive oncologic resections. Though less frequently required in pure gynecologic cases, it has been described in the context of advanced cytoreductive or exenterative surgery [37,39].

It is important to note that these categories are not mutually exclusive; clinical practice often demands their combined use, depending on the extent and location of bleeding. For instance, simultaneous vaginal and pelvic packing may be necessary after radical hysterectomy complicated by concurrent vaginal cuff and retroperitoneal hemorrhage [9]. Furthermore, evolving hemostatic strategies have led to modifications of classical techniques. The integration of balloon tamponade into uterovaginal management reflects a shift toward minimally invasive, monitored, and reversible interventions, reducing reliance on traditional gauze packing [47].

Even in minimally invasive surgery, localized laparoscopic packing—such as the insertion of hemostatic sponges through a 10 mm trocar port—has been described for controlling focal bleeding, particularly during laparoscopic management of ectopic pregnancies or adhesiolysis [45]. While limited by access and challenges in retrieval, these techniques illustrate the adaptability of damage control principles to modern surgical platforms.

Regardless of type, all intra-abdominal or pelvic packs must be meticulously counted, clearly marked (e.g., with radiopaque strips and external tags), and scheduled for removal within 24 to 48 h to minimize risks of infection, compartment syndrome, or retained foreign body [10].

## 5. Decision-Making, Indications, and Contraindications for DCS in OB/GYN

While the principles of DCS are derived from trauma surgery, their application in this specialty is determined by unique physiological states—such as pregnancy-induced hyperdynamic circulation or distorted coagulation in oncological cases—and procedural complexities, including oncologic dissection or obstetrical emergencies. The gravid and recently gravid uterus creates a unique anatomical environment with increased vascularity and altered tissue planes. Physiological adaptations of pregnancy include an increase in blood volume of 30–50%, an elevated cardiac output, and a hypercoagulable state [74]. These factors necessitate modified resuscitation targets and different intervention thresholds. Additionally, bleeding patterns in OB/GYN differ often from trauma: lower-energy injuries compared to trauma, smaller-caliber vessels in many scenarios, and surgical fields altered by pregnancy or tumor anatomy create distinct challenges.

### 5.1. Obstetric Indications

Life-threatening hemorrhage in obstetrics is caused by placental disorders, uterine atony, or traumatic injury, particularly when complicated by coagulopathy or delayed diagnosis. The most common scenarios are

- Placenta accreta spectrum (PAS) with pelvic sidewall/bladder involvement; massive bleeding during/after delivery or hysterectomy [6,40,41,60,75].
- Surgical treatment of refractory uterine atony after failure of uterotonics, balloon tamponade, compression sutures; evolving coagulopathy [2,47].
- Traumatic obstetric hemorrhage (uterine rupture; cervical/vaginal lacerations) following vaginal delivery, especially with delayed diagnosis or transfer [2,7,27,44].
- HELLP-related hepatic rupture (subcapsular or intrahepatic) in unstable patients [76,77].
- Post-massive transfusion coagulopathy with expanding broad-ligament or retroperitoneal hematomas after cesarean delivery [9,28].
- Resource-limited settings lacking blood products or interventional radiology [4].

### 5.2. Gynecological Indications

In gynecology, DCS is most frequently considered during complex oncologic or reoperative procedures, where anatomical distortion and vascular density increase bleeding risk, as well as for potentially catastrophic injuries to major vessels during routine procedures. Examples include

- Cytoreductive surgery for ovarian cancer with extensive pelvic/upper abdominal dissection or pre-existing coagulopathy [9,30].
- Pelvic exenteration with injury risk to presacral venous plexus/internal iliac branches [11,18,19,78].
- Retroperitoneal/presacral bleeding during lymphadenectomy (standard component of many oncologic surgeries) [26,79,80].
- Hemorrhage from tumor-related neovascularization (fragile, anatomically unpredictable vessels) or anatomical variants [79,80].
- Complex pelvic reconstructive surgery (sacrocolpopexy, mesh revision, fistula repair) in scarred/altered planes [12,32].
- Laparoscopic entry vascular injury (iliac or inferior epigastric vessels); incidence 0.04–0.5% [12].
- Vascular injury or diffuse hypervascular oozing in a “frozen pelvis” from endometriosis, infection, or prior surgery [29].
- Acute bleeding from ruptured ovarian cyst/tumor [81].
- Ruptured ectopic pregnancy, especially with delayed diagnosis or rare (e.g., abdominal) presentation [54,55,82].

### 5.3. Clinical Decision-Making and Contraindications for DCS and APP

The decision to perform DCS and APP should be rapid and consider the surgeon’s individual experience (e.g., with internal iliac artery ligation) and the institution’s access to emergent non-surgical procedures (e.g., endovascular embolization) [4,58,60]. Potential (absolute or relative) contraindications to DCS and packing include the following:

- Non-survivable injuries (e.g., non-survivable traumatic brain injury, metastatic disease with catastrophic bleed) or patient-specific factors such as advance directives favoring comfort care. DCS is a life-preserving strategy, not a substitute for thoughtful goals-of-care discussions.
- Uncontrolled bleeding from major central vessels (e.g., abdominal aorta, inferior vena cava) where effective tamponade cannot be achieved with packing alone.
- Uncontrolled infection in the operative field (e.g., perforated viscus with purulent contamination) that may preclude temporary closure and increase sepsis risk.
- Abdominal compartment syndrome (ACS) *without* a decompression plan, as packing can exacerbate intra-abdominal hypertension.

## 6. Evidence-Based DCS Protocol in OB/GYN

Damage control surgery (DCS) in obstetrics and gynecology should follow a structured, multi-phase approach designed to prioritize physiological stabilization over anatomical repair. Originally conceptualized by Rotondo et al. as a three-phase model—abbreviated laparotomy (Phase I), ICU resuscitation (Phase II), and planned re-laparotomy for definitive reconstruction (Phase III)—the framework has evolved to include preoperative resuscitation (Phase 0/Ground Zero) and a dedicated phase for abdominal wall closure, reflecting advances in critical care and open abdomen management [20,23,25].

**Figure 1:**
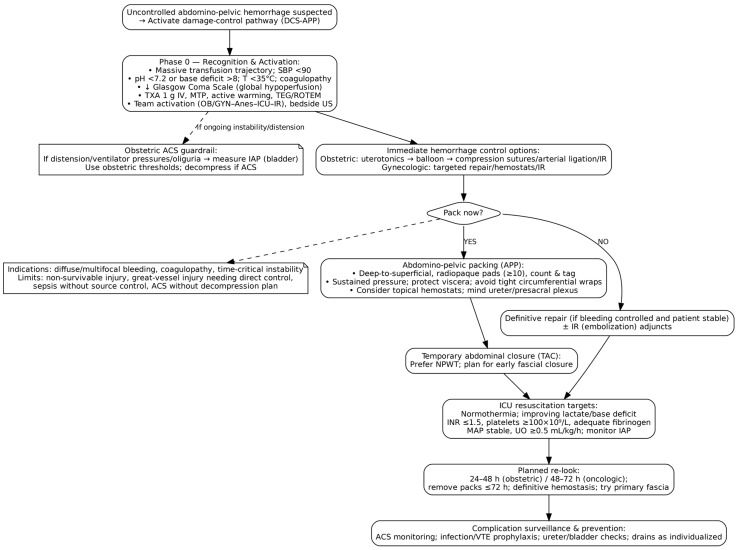
Damage control pathway for uncontrolled abdomino-pelvic hemorrhage in OB/GYN.

This updated protocol—adapted from trauma surgery but refined for specialty-specific contexts—comprises four sequential phases: preoperative resuscitation (Phase 0/Ground Zero), abbreviated surgical control (Phase I), intensive care unit (ICU)-based resuscitation (Phase II), and planned re-laparotomy for definitive reconstruction (Phase III). Each phase is time-sensitive and interdependent, requiring coordinated multidisciplinary action. Figure 1 summarizes the OB/GYN damage control pathway with abdomino-pelvic packing (DCS-APP) from activation through ICU targets and planned re-look.

### 6.1. Phase 0/Ground Zero: Preoperative Resuscitation and Team Activation

Effective DCS begins before the operating room. Early interventions in the emergency department or operating suite are critical to mitigate the lethal triad and prepare for surgical control.

#### 6.1.1. Multidisciplinary Team Assembly

The success of DCS is enhanced by a coordinated, multidisciplinary approach. In high-resource settings, particularly for complex cases like placenta accreta spectrum (PAS), outcomes improve when the team includes a gynecologic oncologist (for retroperitoneal expertise), interventional radiologist, anesthesiologist experienced in massive transfusion, and blood bank coordination [24,28,40,83]. Ideally, a urologist or colorectal surgeon should be available if genitourinary or bowel injury is anticipated, and a hepatobiliary surgeon may be considered in cases of hepatic rupture due to pre-eclampsia [83]. However, in many clinical settings—especially outside tertiary centers—such specialists may not be immediately available.

In these situations, familiarity with the core principles of DCS becomes crucial: rapid hemorrhage control, abbreviated surgery, and physiological stabilization. Early, protocol-driven use of non-surgical hemostatic adjuncts—such as TXA, topical hemostatic agents, and structured blood product resuscitation—can bridge the gap until definitive care is possible. For gynecologic oncology cases, the team is often assembled intraoperatively, as DCS is typically unplanned and arises during procedures with unexpected bleeding [84].

#### 6.1.2. Hemostatic Optimization

Immediate administration of TXA—1 g intravenous over 10 min, ideally within 1 h and no later than 3 h of hemorrhage onset—is strongly supported by the WOMAN trial [49,50,51]. Concurrent activation of a massive transfusion protocol (MTP), targeting a 1:1:1 ratio of packed red blood cells (pRBC), fresh frozen plasma (FFP), and platelets, is essential to pre-empt coagulopathy [28,84]. Administration of 1 g calcium chloride IV (or ~3 g calcium gluconate) during or immediately after the first unit of blood product and after every ~4 units thereafter, with ionized calcium-guided redosing to avoid hypocalcemia-related myocardial dysfunction and coagulopathy [85,86,87].

#### 6.1.3. Volume and Pressure Management

Early resuscitation increasingly follows a restricted fluid strategy to balance perfusion with hemostasis: small crystalloid aliquots act as a bridge to early blood products, avoiding dilutional coagulopathy, edema, and clot disruption [69,88]. Permissive hypotension—maintaining blood pressure below normal until hemostasis—is commonly framed as targeting an SBP around 80–90 mmHg or a MAP in the 50–60 mmHg range in actively bleeding patients without traumatic brain injury (TBI), although evidence is inconsistent and patient-specific [88]. This approach is contraindicated in suspected or confirmed TBI, where higher pressures are required; current recommendations keep SBP ≥ 100–110 mmHg (age-dependent) and MAP ≥ 80 mmHg to preserve cerebral perfusion [69]. When targets are not met with limited fluids and blood products, norepinephrine is frequently used to support MAP while hemostasis proceeds [69]. Supplemental oxygen and monitoring of lactate/base deficit and urine output help assess perfusion adequacy during this controlled resuscitation phase [69,88].

#### 6.1.4. Temperature Management

Active warming (forced-air blankets), warmed IV fluids/irrigants, and room temperature control should start immediately because hypothermia (<35 °C) worsens coagulopathy and is linked to higher mortality in bleeding/trauma patients [68]. Core temperature should be continuously monitored (esophageal, bladder, or pulmonary artery probe). These actions align with contemporary trauma/bleeding guidelines and perioperative evidence showing that even mild hypothermia increases blood loss and transfusion needs, so maintaining normothermia is crucial [69,70].

#### 6.1.5. Laboratory and Point-of-Care Monitoring

Initial assessment should include CBC, a basic metabolic panel, coagulation studies (PT/INR, aPTT), fibrinogen, and type and crossmatch (ensuring adequate pRBC availability). Where available, viscoelastic testing (TEG/ROTEM) provides real-time clot assessment and can guide targeted hemostatic therapy (e.g., fibrinogen/PCC/platelets) in major hemorrhage, including postpartum hemorrhage; if this is not available, clinicians should rely on clinical trajectory and early empiric correction while standard labs are pending [69,89,90].

### 6.2. Phase I: Abbreviated Surgical Control and Packing

The goal of Phase I is rapid hemorrhage control with minimal physiological insult. Definitive repair is deferred.

#### 6.2.1. Surgical Access and Exploration

A vertical midline laparotomy—from the pubis to above the umbilicus, extending to the xiphoid if necessary—is the standard approach, regardless of prior incisions. It provides optimal pelvic access and facilitates packing. A rapid four-quadrant exploration is performed to identify bleeding sources. Obvious injuries are controlled with clamps, sutures, or vessel loops [25].

#### 6.2.2. Contamination Control

In trauma, hollow viscus injuries require control via simple suture repair or stapling; anastomoses and stomas are deferred. However, visceral contamination is rare in obstetric and gynecologic DCS, where hemorrhage—not perforation—is the primary concern [10].

#### 6.2.3. Decision to Pack vs. Repair

Common packing indications are

- Multiple or diffuse bleeding sites (presacral venous plexus, parametrial oozing);
- Hemodynamic instability unlikely to tolerate prolonged surgery;
- Coagulopathy limiting surgical hemostasis;
- Anatomically challenging bleeding areas (retroperitoneum, deep pelvis);
- Persistent bleeding after hysterectomy or internal iliac artery ligation;
- Liver rupture when suturing fails (combined with the Pringle maneuver) [76,77].

#### 6.2.4. Modern Packing Technique

- Material selection: Use large, radiopaque laparotomy pads (minimum 7–10, preferably 10–12). Smaller pads risk being lost during removal, especially after 48 h.
- Pad preparation: Pads may be rolled before placement to create focused pressure. They can be pre-soaked in TXA (100 mg/mL) or coated with a hemostatic agent to enhance local hemostasis [2,27,44,56].
- Placement technique: Pack from deepest to most superficial, applying continuous manual pressure for at least 5 min. Avoid “dead spaces” by overlapping pads tightly—gaps may compromise tamponade [10,37].
- Accountability: Pads should be tied together and strictly counted to ensure complete retrieval.
- Visceral protection: Interpose bowel bags between packing and viscera to prevent adhesions. Consider omentum interposition over raw surfaces when feasible. Ureters should be identified or presumed present in high-risk zones [29].

#### 6.2.5. Temporary Abdominal Closure

Fascial closure is avoided during Phase I of damage control surgery to prevent abdominal wall tension and the development of abdominal compartment syndrome. Instead, a temporary abdominal closure (TAC) technique is employed to contain the viscera, minimize fluid and heat loss, allow for re-exploration, and support physiological recovery. The choice of TAC should be guided by patient stability, anticipated need for re-laparotomy, infection risk, and resource availability. TAC techniques, recently reviewed in [91], comprise the following:

- Skin closure with clips or sutures: Rapid, inexpensive, and easily available, but associated with a high risk of evisceration, infection, recurrent intra-abdominal hypertension, and poor outcomes. These techniques are no longer favored due to high complication and mortality rates.
- Mesh closure and dynamic retention sutures: Meshes (absorbable or non-absorbable) provide a barrier and allow gradual fascial closure but carry a risk of hernia, infection, and adhesions. Dynamic retention sutures help prevent fascial retraction, supporting delayed closure, but are technically demanding and associated with high hernia risk.
- Wittmann Patch (Artificial Burr): This Velcro-like patch enables progressive, staged closure of the abdomen with high rates of primary fascial closure (75–90%). It facilitates repeated re-entries, preserves domain, and is particularly helpful for patients needing multiple operations, though it is costly and does not effectively evacuate peritoneal fluid.
- Bogota Bag: Inexpensive and quickly applied using a sterile irrigation bag sewn to the fascia or skin, it serves as a “non-traction” technique suitable for resource-poor settings. However, it can permit fascial retraction and loss of domain, carries a higher risk of infection and fistula, and is associated with lower closure rates.
- Barker Vacuum Pack: This involves covering the viscera with a polyethylene sheet, then towels and an adhesive drape, and using a suction drain for negative pressure. It is simple and affordable, and, while still used in resource-limited settings, has been largely surpassed by commercial NPT systems because of inferior outcomes in terms of closure rates and complications.
- Commercial Negative Pressure Therapy (NPT) Systems: Devices like 3M™ AbThera™ Therapy are specifically designed for open abdomen management. These systems provide continuous negative pressure, facilitate fluid removal, preserve abdominal domain, and promote fascial approximation. They offer superior closure rates, reduced mortality, and better clinical outcomes; thus, they are the preferred technique according to most guidelines when available, though cost and access may be limiting factors [91].

Abdominal drain placement remains controversial—some advocate for monitoring output, others warn of increased compartment syndrome risk [10,25,27].

### 6.3. Phase II: Critical Care Resuscitation

Postoperative management in the ICU focuses on reversing physiological derangements and preparing for reoperation.

#### 6.3.1. Primary Resuscitation Targets

- Temperature: Maintaining core > 36 °C with active warming.
- Acid–base balance: Targeting pH > 7.25 and base deficit < 4 mEq/L. Avoidance of routine bicarbonate use unless pH < 7.1.
- Coagulation: Target values for fibrinogen are >200 mg/dL (supplement with fibrinogen concentrate if <150 mg/dL), PT/PTT < 1.5× normal, and platelets > 75,000/μL [72].
- Continuation of TXA: 1 g every 8 h should be continued in ongoing bleeding [49,50,51,52].

#### 6.3.2. Monitoring and Complication Prevention

- Abdominal compartment syndrome (ACS): Monitor intra-abdominal pressure (IAP) via bladder catheter. Thresholds: <15 mmHg in non-pregnant, <25 mmHg in peripartum patients [92,93]. Clinical signs include oliguria, hypotension, and elevated peak airway pressures.
- Drain output: Should decrease to <200 mL/hour after coagulopathy correction. Re-laparotomy may be indicated if output exceeds 400 mL/hour in a coagulopathic patient [25].
- Neurological and pain management: Adequate sedation and analgesia are essential, particularly in prolonged ICU stays.
- Enteral feeding: Early enteral nutrition (EN) is associated with fewer infections and higher fascial closure rates [94]. Parenteral nutrition should be initiated started promptly when EN is not feasible. As soon as hemodynamic resuscitation is largely complete and intestinal viability is confirmed, EN should be initiated. A relative contraindication to EN is a viable bowel remnant < 75 cm [95].
- Thromboprophylaxis: Mechanical prophylaxis during coagulopathy; pharmacological after correction [72].
- Antibiotic prophylaxis: In trauma, broad-spectrum antibiotics are standard due to hollow viscus injury. In OB/GYN, an optimal antibiotic prophylaxis is debatable. Usually, second-generation cephalosporins +/− metronidazole are recommended until definitive repair; aminoglycosides should be avoided due to nephrotoxicity and ototoxity [27].

### 6.4. Phase II: Definitive Reconstruction

Timely return to the operating theater is essential to minimize infection risk while ensuring physiological readiness.

#### 6.4.1. Timing of Re-Laparotomy

- Obstetric cases: 24–48 h, as bleeding is typically venous or capillary and stabilizes earlier [7].
- Oncologic or trauma cases: 48–72 h, allowing time for resuscitation and resolution of coagulopathy [10,78].
- The timeframe of 72 h should never be exceeded, as infection risk increases exponentially beyond this point.

#### 6.4.2. Readiness Criteria

Definitive repair should be proceeded when ALL (or the great majority) of the following are satisfied and trending towards normalization:

- Temperature: core temperature ≥ 36 °C (normothermia achieved). Earlier recommendation for at least >6 h at >36 °C seems arbitrary; trend and stability matter more than time box [96].
- Acid–base and perfusion: lactate falling (preferably toward <2–3 mmol/L or clear downward trend) and base deficit improving (e.g., >−6 to −5 and rising toward 0) [69,97].
- Coagulation: INR ≤ 1.5, platelets ≥ 100 × 10^9^/L, fibrinogen adequate (many centers target ≥ 2.0 g/L), with no active diffuse coagulopathic oozing [69].
- Hemodynamics: stable MAP with minimal or no vasopressor support and adequate urine output (≥0.5 mL/kg/h), indicating restored end-organ perfusion. IAP < 15 mmHg is a reasonable safety boundary for closure (IAH is ≥12 mmHg; ACS is >20 mmHg with new organ dysfunction) but should be combined with overall abdominal compliance/closure feasibility [69].
- Abdominal domain/compliance: abdomen amenable to safe closure without provoking intra-abdominal hypertension (IAP kept < 12–15 mmHg) OR an open-abdomen plan is in place (NPWT with planned staged closure) [92,95,98].
- Transfusion trajectory: no ongoing massive transfusion requirement and blood product use clearly decelerating (many teams use “≤2 units over 4–6 h” pragmatically, though not formally guideline-mandated) [69].
- There is no validated abdominal drain-output threshold, which is congruent with overall weak evidence for routine drainage in surgery. Some sources recommend removing drains when output is <200 mL/h, but the individual threshold should be seen in context of injury pattern, TAC technique, patient hemodynamics, and feasibility of safe closure instead [78,95].

#### 6.4.3. Pack Removal and Definitive Hemostasis

- Preparation: Full surgical team, anesthesia with muscle relaxation, blood products at bedside, and setup for re-packing if needed.
- Technique: Irrigate the field with warm saline before removal. Remove packs slowly and systematically, applying counter-pressure. Inspect each anatomical zone for residual bleeding.
- Definitive control: Address active bleeding with sutures, clips, or vessel ligation. Use hemostatic agents as adjuncts.
- Closure: After copious irrigation, attempt primary fascial closure if possible—achieved in 60–80% of cases. If not, use NPT or planned skin grafting.

## 7. Complications and Outcomes of DCS in OB/GYN

DCS with pelvic packing is a life-saving intervention, but it carries significant risks arising from the physiological stress of hemorrhage, the surgical technique itself, and prolonged critical illness [27]. Understanding these risks and implementing preventive strategies is essential for optimizing outcomes in obstetric and gynecologic patients. The most common complications and mitigation strategies are summarized in Table 1. Prevention, diagnostic, and management options are listed in Table 2.

### 7.1. Abdominal Compartment Syndrome (ACS)

Abdominal compartment syndrome (ACS) is the most serious specific complication of damage control surgery (DCS), occurring in 10–40% of patients who undergo the procedure [92,99]. ACS is a life-threatening condition in critically injured patients caused by increased intra-abdominal pressure (IAP), which can lead to multiorgan failure. Elevated IAP compromises perfusion to vital organs, resulting in cardiovascular, respiratory, renal, and neurological dysfunction, which may culminate in left ventricular failure. Normal intra-abdominal pressure ranges from 5 to 7 mmHg and may rise to 10–11 mmHg in critically ill, non-pregnant patients [92]. According to the World Society of the Abdominal Compartment Syndrome (WSACS), intra-abdominal hypertension (IAH) is defined as a sustained IAP ≥ 12 mmHg, while ACS is diagnosed when IAP exceeds 20 mmHg with new-onset organ dysfunction [92].

However, in pregnancy, baseline IAP is physiologically elevated due to the expanding uterus, amniotic fluid, and placenta. During the third trimester, median IAP ranges from 15 to 29 mmHg, and it decreases to approximately 16 mmHg within 24 h postpartum [93,98,100,101]. Pregnant women adapt to this increase (as a result of growing fetus/es, amniotic fluid and placenta) by developing greater abdominal wall compliance [98,100,101]. Therefore, as pregnant or postpartum patients have an elevated baseline IAP, diagnostic thresholds derived from non-pregnant populations are not directly applicable to obstetric and postpartum patients. Clinical signs—such as hypotension, oliguria, abdominal distension, and elevated peak airway pressures—should be integrated with IAP measurements for accurate diagnosis [27,98,101].

Additional conditions in pregnancy—such as pre-eclampsia, eclampsia, and HELLP syndrome—can further elevate IAP [98]. In the context of an open abdomen, excessive or tightly packing may also contribute to ACS, particularly when combined with fluid overload or bowel distension. Gynecologic conditions associated with increased IAP include large retroperitoneal hematomas, ovarian hyperstimulation syndrome, malignant ascites, and prolonged laparoscopy with high insufflation pressures, among others [98].

Typical ACS symptoms include hypotension, oliguria, abdominal distension, and elevated ventilator peak pressures. Diagnosis of IAP is most reliably assessed via bladder pressure measurement, the gold standard recommended by WSACS. A Foley catheter is used to instill 25 mL of sterile saline, and pressure is measured at the mid-axillary line using a manometer. In older literature, gastric pressure via a nasogastric (Ryle’s) tube connected to a water manometer was considered an alternative [23,27,92].

Treatment depends on severity and clinical stability:

- Non-surgical measures: nasogastric or rectal decompression, gastrointestinal prokinetics, diuretics, and neuromuscular blockade to reduce intra-abdominal volume and abdominal wall tone.
- Surgical decompression: indicated when medical management fails and organ dysfunction persists; may involve opening a closed fascia, loosening or removing packs (with potential risk of re-bleeding), or extending the abdominal incision [27].

### 7.2. Infectious Complications

A significant proportion of patients undergoing APP develop febrile morbidity. The most common non-specific infectious complications are intra-abdominal (pelvic, subphrenic, intramesenteric) abscesses and sepsis. In trauma surgery, the incidence of intra-abdominal abscesses is consistently reported as high, due to a combination of contaminated operative fields, the presence of foreign material in the abdominal cavity, shock, and immunocompromised patients [21]. For OB/GYN DCS, data on infection-related morbidity after APP are conflicting. While some authors report “near-universal febrile morbidity” [36], others report lower rates of infection-related complications. In OB/GYN series comparing APP with no APP for intractable hemorrhage, infection rates were similar, while bleeding control was significantly higher with APP [9,41].

Given the variable infection rates reported with APP, broad-spectrum antibiotic prophylaxis is recommended [41]. Prolonged packing (>72 h) remains a major risk factor for infectious morbidity [32]; therefore, early removal of packing materials—24–48 h in obstetric cases and 48–72 h in oncologic cases—is mandatory [7,32,41].

### 7.3. Urological Complications

The ureters are most vulnerable to compression at two anatomical sites: the pelvic brim where they pass under the uterine artery and the ureterovesical junction near the bladder [29,102]. Clinical signs of ureteral compromise typically emerge 24–48 h postoperatively and include oliguria, rising serum creatinine, and hydronephrosis on imaging [12]. In prolonged compression related to extraperitoneal pelvic packing, renal pelvicalyceal rupture has been reported [103]. When ureteral injury or obstruction is suspected, management requires prompt decompression, e.g., by loosening or repositioning packs or ureteral stenting, or—if stenting fails—percutaneous nephrostomy to preserve renal function and prevent permanent damage [12].

### 7.4. Vascular Complications

Vascular complications arise primarily from compression of major pelvic vessels during packing, leading to deep vein thrombosis and potential limb ischemia [41,104]. Among trauma patients with isolated severe pelvic fractures, those treated with preperitoneal pelvic packing had significantly higher venous thromboembolism rates and worse survival than matched patients managed without packing [105]. Inadvertent ligation or injury of major vessels during pre-packing maneuvers can result in large-territory ischemia (e.g., limb hypoperfusion, mesenteric/colonic ischemia) or severe venous outflow obstruction [104,106,107]. Accordingly, detailed knowledge of pelvic vascular anatomy and common variants is crucial for preventing and managing severe pelvic bleeding [58,79,80]. This anatomic familiarity is particularly important in complex oncologic operations and emergencies, where rapid orientation is essential for effective, atraumatic pack placement [58]. Once hemostasis is achieved and bleeding risk declines, pharmacologic thromboprophylaxis should be initiated promptly to prevent thromboembolic events. Continuous limb-perfusion monitoring (pulses/Doppler, capillary refill, limb temperature) is essential. If vascular compromise is suspected, urgent vascular surgery consultation is warranted to prevent irreversible ischemic injury or limb loss [104,107,108].

### 7.5. Outcomes of APP/DCS

#### 7.5.1. Short-Term Outcomes

Contemporary series demonstrate favorable outcomes when DCS is appropriately applied in obstetric and gynecologic emergencies. Hemostasis is achieved in 62–90% of patients, with reports of substantially lower mortality versus conservative management alone [5,7,9,16]. When performed within appropriate timeframes, overall survival exceeds 80%, and primary fascial closure is achieved at re-laparotomy in 60–80% of cases [7,9,27].

In the largest multicenter study of abdominal packing after unsuccessful peripartum hysterectomy, Deffieux et al. reported a 62% success rate (33/53) for hemorrhage control without additional procedures; median pack dwell was 39.5 h (IQR 24–48), and no packing-related infectious complications were reported [7]. Kumar et al. [9] reported an 87.5% success rate, and Yoong et al. [37] reported 100% control of intractable venous hemorrhage with abdomino-pelvic packing in a small series.

#### 7.5.2. Long-Term Outcomes

OB/GYN-specific long-term data after APP/DCS remain sparse, so evidence extrapolated from trauma cohorts should be interpreted with caution. In trauma patients, early fascial closure (≤7 days) is associated with better long-term quality of life and a higher return-to-work rate (54% vs. 10%) compared with delayed closure [109]. Patients managed with damage control strategies have elevated risks of late complications—including ventral hernia, enterocutaneous fistula, intra-abdominal adhesions, bowel obstruction, and reduced SF-36 scores—on long-term follow-up [110,111]. Persistent pain and psychological sequelae are also frequent after major trauma; meta-analytic data in ICU/trauma survivors show PTSD and depressive symptoms in 20–30% at 6–12 months [112]. In obstetrics, a recent systematic review highlights long-term physical, psychological, and social consequences after peripartum hysterectomy, including PTSD, depression, anxiety, and grief; when DCS is required as a salvage measure after failed peripartum hysterectomy, a comparable or greater psychological burden is plausible, underscoring the need for routine psychological screening and early referral to support services [113].

#### 7.5.3. Key Factors for Optimal Outcomes

- Early intervention before the lethal triad (hypothermia, acidosis, coagulopathy) becomes established.
- Appropriate patient selection.
- Multidisciplinary team experience (gynecology/obstetrics, anesthesia/critical care, interventional radiology, vascular/urology).
- Integration of modern hemostatic strategies (TXA, topical hemostats, embolization).
- Strict aseptic technique.
- Timely pack removal (24–48 h, obstetric; 48–72 h, oncologic).
- Comprehensive postoperative monitoring and support (infection surveillance, DVT prophylaxis when safe, nutrition/physiotherapy, psychological support).

### 7.6. Special Considerations in Gynecologic Oncology

In gynecologic malignancy, DCS/APP intersects with risk factors that impair recovery—cachexia, anemia, hypoalbuminemia, and recent chemotherapy—which worsen wound healing and infection risk [18,19]. Early goals are hemostasis, source control, and nutritional/hematologic optimization, followed by timely DVT prophylaxis once bleeding risk allows. Multidisciplinary planning should define criteria and timing to resume chemotherapy (after pack removal and stable wound healing) to avoid compromising oncologic control [9,17]. This targeted approach preserves the hemostatic benefits of DCS while minimizing delays in adjuvant therapy.

## 8. Discussion

The roots of APP within the DCS framework lie in late-20th-century trauma surgery, when rapid tamponade of exsanguinating pelvic or retroperitoneal hemorrhage was often the only temporizing option [10]. Although much procedural knowledge derives from the trauma literature, direct transposition to contemporary OB/GYN practice is limited by anatomic, physiologic, and situational differences [27,73,98]. Trauma patients commonly present with complex pelvic fractures, high-energy blunt injuries, and multisystem damage. In contrast, obstetric scenarios more often involve iatrogenic vessel injury in otherwise healthy patients or hemorrhage from gravid or recently gravid uteri. Physiologic adaptations of pregnancy—including increased blood volume, cardiac output, and baseline intra-abdominal pressure—affect both presentation and resuscitation targets in hemorrhagic shock [98], necessitating modified thresholds for intervention. In gynecologic oncology, cytoreductive surgery, e.g., for advanced ovarian cancer, entails large-volume resections, difficult-to-access regions, and retroperitoneal and upper-abdominal dissections, with risks of major vessel injury and broad raw surfaces prone to diffuse oozing [9,10,11,114,115]. Tumor-related coagulopathy, extensive surgical trauma, and compromised nutritional status further complicate hemostasis [30,96]. In benign gynecology, packing may be life-saving in rare but critical scenarios such as vascular injury during laparoscopic trocar insertion or intense bleeding from pelvic vessels during adhesiolysis or deep endometriosis surgery [12,29,56]. Catastrophic intra-abdominal bleeding can also be triggered by seemingly atraumatic circumstances like consensual intercourse [14,15,16].

The risk–benefit profile of pelvic packing in OB/GYN remains incompletely defined due to a paucity of large, controlled studies. Available data suggest high immediate hemostatic success (75–90%), tempered by risks of infection, organ compression, and thrombosis [26]. Meticulous technique, anatomic familiarity, appropriate material selection, and strict timelines for pack removal (24–48 h for obstetric cases; 48–72 h for oncologic cases) are central to minimizing these risks [78]. Whenever possible, APP duration should not exceed these limits. Understanding pelvic vascular anatomy and recognizing difficult-to-access areas are crucial for preventing and managing major pelvic hemorrhage [79,80]. This knowledge is particularly important during complex oncologic procedures, where variant vascular patterns are common, and in emergencies, where rapid orientation is essential for effective, atraumatic pack placement [114,115].

Advances in adjunctive measures have shifted pelvic packing from a last resort to an integrated component of staged hemostatic control. Early TXA provides survival benefit in trauma (CRASH-2) and postpartum hemorrhage (WOMAN) [49,50,51,52,116] and is increasingly adopted in major gynecologic surgery. Maternal mortality disproportionately affects low- and middle-income countries, where blood products, ICU capacity, and specialized expertise may be limited [1,116,117]. TXA’s heat-stable, low-cost profile is valuable in such settings and may reduce reliance on resource-intensive interventions [116].

Topical hemostats—including flowable gelatin–thrombin matrices, fibrin sealants, oxidized regenerated cellulose, and chitosan-based dressings—offer targeted control of parenchymal or difficult-to-access bleeding [30,44,55,56]. In oncologic surgery, early application can prevent progression to uncontrolled hemorrhage and reduce the need for packing or reoperation [3]. Use of agents containing coagulation components (fibrin, thrombin) reduces operative time, blood loss, and transfusion needs—even in patients with disturbed coagulation—potentially lowering the need for DCS [30]. Beyond pre-APP use, hemostatic agents have been applied to soak/coat packing materials [2].

Multiple reports support APP/DCS in OB/GYN. In placenta accreta spectrum, pelvic packing controlled persistent bleeding after peripartum hysterectomy without postoperative complications in a small series [55]. A modified “pelvic pressure packing” using a Foley-condom saline device (removed at 48–72 h) achieved definitive hemostasis in 11 obstetric/gynecologic cases with no morbidity or mortality [1]. APP also controlled massive bleeding after placental removal in abdominal pregnancy [56]. In the largest multicenter cohort of packing after unsuccessful peripartum hysterectomy, the success rate was 62%, with an overall mortality of 24%, reflecting illness severity rather than the packing itself [5]. A 6-patient series of “Karateke packing” (near-hot sponges wrapped around a Bakri balloon, 500–1000 mL, vaginal traction) achieved hemostasis in 5/6; 1 patient with placenta percreta died from rupture and hypovolemic shock; no packing-related complications were reported among survivors [61]. In gynecologic oncology, combined intra-abdominal/intrapelvic packing during cytoreductive surgery for advanced ovarian cancer showed 12.5% operative mortality (2/16) and no excess morbidity versus controls [4]. After failure of internal iliac ligation, a ribbon-gauze/Penrose drain pack arrested bleeding in four additional cases (one postpartum, three oncologic) [2].

Despite its life-saving potential, APP’s effectiveness depends on team readiness and institutional preparedness. Because its use is infrequent, competency should be maintained through structured programs—simulation-based education, multidisciplinary crisis drills, and mentorship in high-volume centers [118]. In resource-limited settings where embolization is unavailable, packing remains a low-cost, high-impact option. Emphasis should be placed on early TXA, basic packing techniques with available materials, and regular training [118,119]. Cadaver-based courses in Scandinavia demonstrated that formal training increases both frequency and accuracy of packing, confirming that the skill is teachable and sustainable [119]. For OB/GYN, integrating DCS principles into residency curricula and hospital massive hemorrhage protocols is essential, keeping in mind that intractable hemorrhage can occur at any time and in any setting, especially in obstetric departments. Although previous PPH, pre-existing anemia, chorioamnionitis, fetal macrosomia, multiple gestation, pre-eclampsia, large uterine myomas, prior uterine surgery, or obesity are well-known risk factors for PPH related to the “four Ts”—trauma of the birth canal, (intrauterine) tissue retention, atony, and thrombin alterations (coagulopathy)—in most cases (ca. 60%), PPH occurs without considerable risk factors [117]. In the near future, AI-supported training platforms should improve training in DCS and emergency protocols, by including real-time “unpredictable” scenarios and reflecting the tempo and uncertainty of real emergencies. High-fidelity virtual reality/augmented reality simulation and telementoring can rehearse rare bleeding patterns, equipment failures, and human factor stressors under time pressure, with objective metrics and debriefs [120,121].

In this review, we analyzed the quasi “linear” maturation of DCS, from its first definitions and conceptualizations to progressive enrichment with pharmacologic adjuncts (e.g., TXA, topical hemostats) and standardized blood-management protocols. We hypothesize that the next decade will be driven by AI- and robot-supported protocols, which can synchronize early diagnosis and protocolized laboratory and imaging work-ups with automatic multidisciplinary activation within minutes. Currently, we observe an emerging shift from “robotic-assisted” to “robotic-guided” surgery, as artificial intelligence helps fuse augmented reality and multimodal inputs (imaging, radiomics, molecular diagnostics) into a visually and informationally enhanced field [122]. Ideally, personalized DCS should start before the incision. AI can fuse patient-specific profiles—comorbidities, anticoagulants, coagulation phenotype, prior hemorrhage, and even genetic markers—to stratify bleeding risk, pre-position resources, and lower the threshold for an abbreviated, packing-first strategy when the risk of damage extension is high [123]. Intraoperatively, robotic techniques can be paired with 3-D reconstructions, AR overlays, fluorescence imaging, and AI decision-support, providing real-time alerts (e.g., impending hypotension) and guiding strategies (e.g., packing vs. definitive repair), highlighting anatomic danger zones, and documenting critical steps, while postoperatively enabling risk-stratified monitoring for rebleeding, ACS, or infection [123,124].

## 9. Conclusions

APP within a DCS strategy is a potentially life-saving option for “near miss” patients with persistent, uncontrollable bleeding. In post-hysterectomy pelvic hemorrhage and selected obstetric and oncologic emergencies, reported success rates are high, with acceptable morbidity. Therefore, DCS principles should be embedded in obstetric hemorrhage guidelines and institutional massive bleeding protocols, with APP retained a defined step for refractory bleeding. Training obstetricians and gynecologists—via simulation and hands-on courses—can reduce delays and improve outcomes. In resource-limited settings, where interventional radiology (arterial embolization) may be unavailable, APP offers a pragmatic, effective bridge to resuscitation and definitive care.

## Figures and Tables

**Table 1:** Complications associated with APP in DCS—an overview.

Category	Complication	Mechanism/Incidence
Abdominal	Abdominal compartment syndrome (ACS)	Elevated IAP from over-packing, edema, or fluid overload; 10–40% in open-abdomen/ DCS cohorts (lower in OB/GYN)
	Lateral fascial retraction	Prolonged open abdomen → abdominal wall retracts and bowel swells (“loss of domain”); complicates delayed closure
	Entero-atmospheric fistula	Exposed bowel + inflammation/infection; rare in OB/GYN
	Fascial dehiscence/evisceration	Edema, infection, poor tissue quality; up to ~10% in open-abdomen series
Urological	Ureteral/bladder compression	Packs/hematoma/edema compress urinary tract; often evident 24–48 h post-op
	Obstructive acute kidney injury	Ureteric kinking/obstruction; bladder outlet compression (esp. with extraperitoneal packing)
	Collecting system rupture (rare)	High back-pressure with extraperitoneal pelvic packing
Vascular	Great-vessel compression (IVC/iliac venous outflow)	Excessive or malpositioned packs → ↓ venous return
	Arterial/venous thrombosis	Vessel injury/ligation + compression; low flow/hypercoagulability
	Rebleeding after pack removal	Incomplete control or arterial source; 5–10% (series-dependent)
Neurological	Peripheral neuropathy (sciatic/femoral/obturator)	Compression, over-packing, prolonged positioning
Gastrointestinal	Mechanical bowel obstruction	Adhesions or extrinsic compression by packs
	Paralytic ileus	Inflammation, opioids, electrolyte disturbance
Pelvic floor related	Rectovaginal fistula (rare)	Tissue ischemia/infection in scarred fields
	Vaginal cuff dehiscence (post-hysterectomy)	Poor tissue quality, infection, tension
	Pelvic floor dysfunction	Nerve/muscle injury, scarring
Infectious	Intra-abdominal abscess	Up to 25% in trauma open-abdomen series; lower in OB/GYN with early pack removal
	Sepsis	Secondary to ongoing contamination/necrosis
	Surgical-site infection	Higher with immunosuppression, transfusion, prolonged open abdomen
Systemic	Pyrexia (post-op)	Inflammatory response; infection must be excluded
	DVT/PE	Immobility, venous injury, hypercoagulability
	Multi-organ failure	Ongoing shock/“lethal triad”

Abbreviations: IAP—intra-abdominal pressure; ACS—abdominal compartment syndrome; NPWT—negative-pressure wound therapy; UO—urine output; IVC—inferior vena cava; DVT—deep vein thrombosis; PE—pulmonary embolism; IR embolization—interventional radiology (transcatheter) embolization.

**Table 2:** Complications of APP: prevention, diagnosis, and management strategies.

Complication	Typical Early Signs	Prevention	Diagnosis/ Monitoring	Management Strategies
Recurrent bleeding/persistent hemorrhage	Ongoing drain output, instability, Hb drop	Early correction of coagulopathy (MTP, fibrinogen/platelets); adequate pad number/depth; avoid tight circumferential wraps	Hemodynamics, Hb/INR/fibrinogen; bedside US/CT if stable	Return to OR for re-packing or targeted hemostasis; correct coagulopathy; consider IR adjuncts (e.g., uterine/internal iliac embolization or temporary balloon occlusion) for a focal arterial source
Bleeding on pack removal	Brisk oozing at re-look	Planned re-look within recommended dwell time; normothermia and corrected labs before removal	Intra-op assessment during re-look	Remove packs sequentially with direct visualization; add hemostats/ligations; re-pack if needed; consider IR embolization for focal bleeding
Infection/intra-abdominal abscess	Fever, leukocytosis, foul drains	Early pack removal (see footnote); sterile technique; minimize devitalized tissue	WBC, CRP; imaging (US/CT) if stable	Culture-directed antibiotics; drain collections (percutaneous if feasible); debridement at re-look if source persists
Surgical-site infection/wound dehiscence	Erythema, discharge, fascial gaping	NPWT/appropriate TAC; avoid excessive moisture; glycemic control	Bedside exam; TAC checks	Open and drain; debride nonviable tissue; optimize TAC; staged closure once clean
Intra-abdominal hypertension/ACS	Rising ventilator pressures, oliguria, tense abdomen	Limit over-packing; avoid tight circumferential pressure; judicious fluids	Measure IAP (bladder); monitor UO and ventilator pressures	Decompress (release tension/adjust packs, reopen TAC); correct drivers (fluid balance); manage per obstetric ACS guardrails
Visceral injury (bowel/mesentery)	Unexpected hemoperitoneum, enteric leakage, peritonitis	Protect viscera with sheets; place pads deep → superficial; avoid shearing	Intra-op inspection at re-look; CT with contrast if stable	Repair primarily if feasible; resection/diversion for non-viable or contaminated injuries; broad-spectrum antibiotics
Enteric fistula (entero-/enterocutaneous)	Persistent enteric drainage, skin irritation	Gentle packing; early re-look; avoid pad edges abrading serosa	Drain amylase/bile; CT with oral contrast if stable	Sepsis control, skin protection, nutritional support (consider TPN); diversion/stoma if needed; delayed reconstruction
Ureteral obstruction/hydronephrosis	Rising creatinine, flank pain (later), low UO	Know ureteral course; avoid medializing pads against ureter	US (hydronephrosis); CT if stable	Reduce/reposition packs; ureteral stent or percutaneous nephrostomy if obstruction persists
Bladder compression/retention or injury	Low UO despite resuscitation, suprapubic distension, hematuria	Avoid pad pressure on bladder dome; ensure Foley patency	Bladder scan/flush; cystography if injury suspected	Reposition packs; ensure Foley function; repair injury at re-look if confirmed
Vascular thrombosis/VTE	Calf swelling, hypoxia	Early mechanical prophylaxis; start pharmacologic prophylaxis once hemostasis secure	D-dimer (limited), duplex/CTPA if clinically indicated	Anticoagulate when safe; IVC filter selectively if anticoagulation contraindicated
Renal impairment (pre-renal/obstructive)	Rising creatinine, oliguria	Goal-directed resuscitation; avoid over-packing near ureters	UO/hour, creatinine; renal US	Optimize volume/pressors; correct ACS/obstruction; renal consult if persistent
Respiratory compromise from packing	Elevated airway pressures, low tidal volumes	Avoid diaphragm-elevating pressure; limit over-packing	Ventilator pressures, ABGs	Lighten/reposition packs; adjust ventilator; treat ACS if present
Retained pack/count discrepancy	Pack count off; radio-opaque marker seen	Strict count and documentation; radio-opaque pads only	Intra-op X-ray if count mismatch	Immediate search; return to OR if not found
Loss of abdominal domain / difficult fascial closure	Progressive fascial retraction	Early plan for closure; NPWT with progressive tension; avoid prolonged dwell	Bedside fascial gap assessment	Progressive closure techniques; later component separation if needed; plastic-surgery consult
Empty pelvis syndrome	Small bowel descent into pelvic dead space → adhesions/obstruction	Omentoplasty or pelvic exclusion techniques when large pelvic dead space anticipated	Clinical signs of obstruction; imaging if stable	Omentoplasty; consider pelvic mesh/sling solutions in selected cases; early recognition and surgical correction if obstructive course

Abbreviations: APP—abdomino-pelvic packing; TAC—temporary abdominal closure; NPWT—negative-pressure wound therapy; IR—interventional radiology; IAP—intra-abdominal pressure; ACS—abdominal compartment syndrome; PCN—percutaneous nephrostomy; VTE—venous thromboembolism; Hb—hemoglobin; US—ultrasound; CT—computed tomography; OR—operating room; MTP—massive transfusion protocol; TPN—total parenteral nutrition.
